# The expression pattern of PFKFB3 enzyme distinguishes between induced-pluripotent stem cells and cancer stem cells

**DOI:** 10.18632/oncotarget.4995

**Published:** 2015-08-13

**Authors:** Artur Cieślar-Pobuda, Mayur Vilas Jain, Gunnar Kratz, Joanna Rzeszowska-Wolny, Saeid Ghavami, Emilia Wiechec

**Affiliations:** ^1^ Department of Clinical and Experimental Medicine, Division of Cell Biology, Linköping University, Linköping, Sweden; ^2^ Integrative Regenerative Medicine Center (IGEN), Linköping University, Linköping, Sweden; ^3^ Institute of Automatic Control, Silesian University of Technology, Akademicka 16, Gliwice, Poland; ^4^ Experimental Plastic Surgery, IKE, Linköping University, Linköping, Sweden; ^5^ Department of Plastic Surgery, County of Östergötland, Linköping, Sweden; ^6^ Department of Human Anatomy and Cell Science, University of Manitoba, Manitoba, Canada

**Keywords:** cancer stem cells, induced pluripotent stem cells, PFKFB3, PFK1, R-point

## Abstract

Induced pluripotent stem cells (iPS) have become crucial in medicine and biology. Several studies indicate their phenotypic similarities with cancer stem cells (CSCs) and a propensity to form tumors. Thus it is desirable to identify a trait which differentiates iPS populations and CSCs. Searching for such a feature, in this work we compare the restriction (R) point-governed regulation of cell cycle progression in different cell types (iPS, cancer, CSC and normal cells) based on the expression profile of 6-phosphofructo-2-kinase/fructose-2,6-biphosphatase3 (PFKFB3) and phosphofructokinase (PFK1). Our study reveals that PFKFB3 and PFK1 expression allows discrimination between iPS and CSCs. Moreover, cancer and iPS cells, when cultured under hypoxic conditions, alter their expression level of PFKFB3 and PFK1 to resemble those in CSCs. We also observed cell type-related differences in response to inhibition of PFKFB3. This possibility to distinguish CSC from iPS cells or non-stem cancer cells by PFKB3 and PFK1 expression improves the outlook for clinical application of stem cell-based therapies and for more precise detection of CSCs.

## INTRODUCTION

Recent advances in the reprogramming field have shown the great usefulness of induced pluripotent stem cells (iPS) in various applications such as drug screening, disease modeling, toxicity testing, gene therapies and regenerative medicine [1]. After a decade of constraints due to ethical issues related to the use of embryonic stem cells (ESC), the creation of functionally similar, induced pluripotent stem cells (iPS) is allowing stem cell biology and regenerative medicine to become a flourishing research area [1, 2]. However, the critical issue hampering progress in the therapeutic use of iPS (and ESC) is the increased propensity of iPS-derived tissues to form teratomas [3, 4]. A crucial event in initiating tumors is activation of the self-renewal machinery, which is normally a characteristic of stem cells. Therefore, it is likely that cancer initiating cells, known as cancer stem cells (CSCs), share molecular signatures detected in iPS cells [5–8]. CSCs are a subgroup of cells within a tumor having the ability of self-renewal and the capability to initiate tumor formation when transplanted, and they comprise a key target of anti-cancer therapies [9, 10]. The identification of CSCs in several human cancers (breast, brain, skin, head & neck, thyroid, cervix, retina, lung, leukemia and lymphoma) provides a new way to understand tumorigenesis at the cellular level [11]. The increased tumor-initiating capability of iPS results from the residual undifferentiated iPS after transplantation or from the use of protooncogenic transcription factors during generation of iPS [12].

Both CSCs and iPS exhibit altered cell cycle regulation as compared to normal cells, but iPS cells are characterized by a relatively short G1 phase and a shortened cell cycle (16–18 h) [13], whereas CSCs, when compared with cancer cells, have a long G2 phase and a higher ratio of cells in G2 phase [14, 15].

The cell cycle is a carefully regulated, finely coordinated process essential for proper functioning of a cell and for reproduction of a whole organism. The decision about cell division is taken on the basis of several factors like the availability of growth factors or cell density [16] and is made during the G1 phase at the so-called restriction (R) point. This G_1_-R-point plays a vital role in the cell cycle since here the external signals (i.e. from growth factors, adhesion molecules and hormones) and metabolic signals (energetic state of the cell, availability of building blocks) converge. Once a cell passes the R-point and commits to the cell cycle, external signals are no longer needed to enter S phase, and cell cycle progression depends largely upon checkpoint regulators [17, 18]. The components and regulation of the G_1_-R point are not fully understood, despite over 20 years of intense research. Recently, the glycolysis-promoting enzyme 6-phosphofructo-2-kinase/fructose-2, 6-bisphosphatase, isoform 3 (PFKFB3) has been identified as one of the hallmarks of the cellular G_1_-R point [19, 20]. While the exact function of PFKFB3 in R-point decision-making is not fully understood, it may serve as an element of a metabolic sensor, which helps to determine whether a sufficient amount of energy is available to enter a new cell cycle. The metabolic properties of normal cells differ considerably from those of cancer cells. In normal cells energy production depends on oxidative metabolism, whereas in the majority of cancer cells (except for diffuse large B-cell lymphoma and glioblastoma) on aerobic glycolysis [21–23]. The increased glucose uptake with concomitant lactate production, even under aerobic conditions, is known as the Warburg effect [24]. However, the role of glucose metabolism in the control of iPS and CSCs remains largely unknown.

PFKFB3 is overexpressed in some tumors [25–28], and its silencing prevents cells from entering the S phase and decreases glucose uptake as well as inducing autophagy in order to decrease tumor burden [19, 29, 30]. The product of PFKFB3, fructose-2,6-biphosphate (F2,6BP), is in turn an allosteric activator of the glycolytic enzyme 6-phosphofructo-1-kinase (PFK1). Tudzarova and coworkers have demonstrated that cells arrested by glucose deprivation can progress into S phase after the replacement of glucose only in the presence of PFKFB3 or upon its replacement by PFK1 [20]. These results suggest that PFKFB3 and PFK1 not only play a pivotal role in glycolysis, but also may be closely associated with the R-point.

The R-point is believed to be disrupted in cancer cells since they do not enter a G0-quiescent phase, but continually re-enter the cell cycle [31]. Like CDK inhibitors, the R-point is considered as a promising target for cancer treatment in order to selectively eliminate only proliferating cancer cells [18]. Furthermore, successful anti-cancer therapy should be aimed primarily at targeting CSCs, which have the ability of self-renewal and the capability to generate diverse tumor cells.

Despite the fact that iPS are phenotypically similar to CSCs, the differences between these cells are not well defined. For many years, the development of clinical tests for CSCs have been hampered by the lack of good methods to distinguish CSCs and normal stem cells. Currently, most CSCs are identified based on cell surface markers or intracellular molecules [32]. Here, we hypothesize that CSCs can be distinguished from iPS or from other cancer cells within the bulk of the tumor on the basis of PFKFB3 and PFK1 expression, and we show the differences between CSCs and iPS in PFKFB3 and PFK1 expression. Since metabolism is becoming an important diagnostic and therapeutic target, characterization of the energetic properties of CSCs is essential.

## RESULTS

### Enrichment of breast cancer cell line-derived mammospheres for cancer stem cells

**Figure 1:**
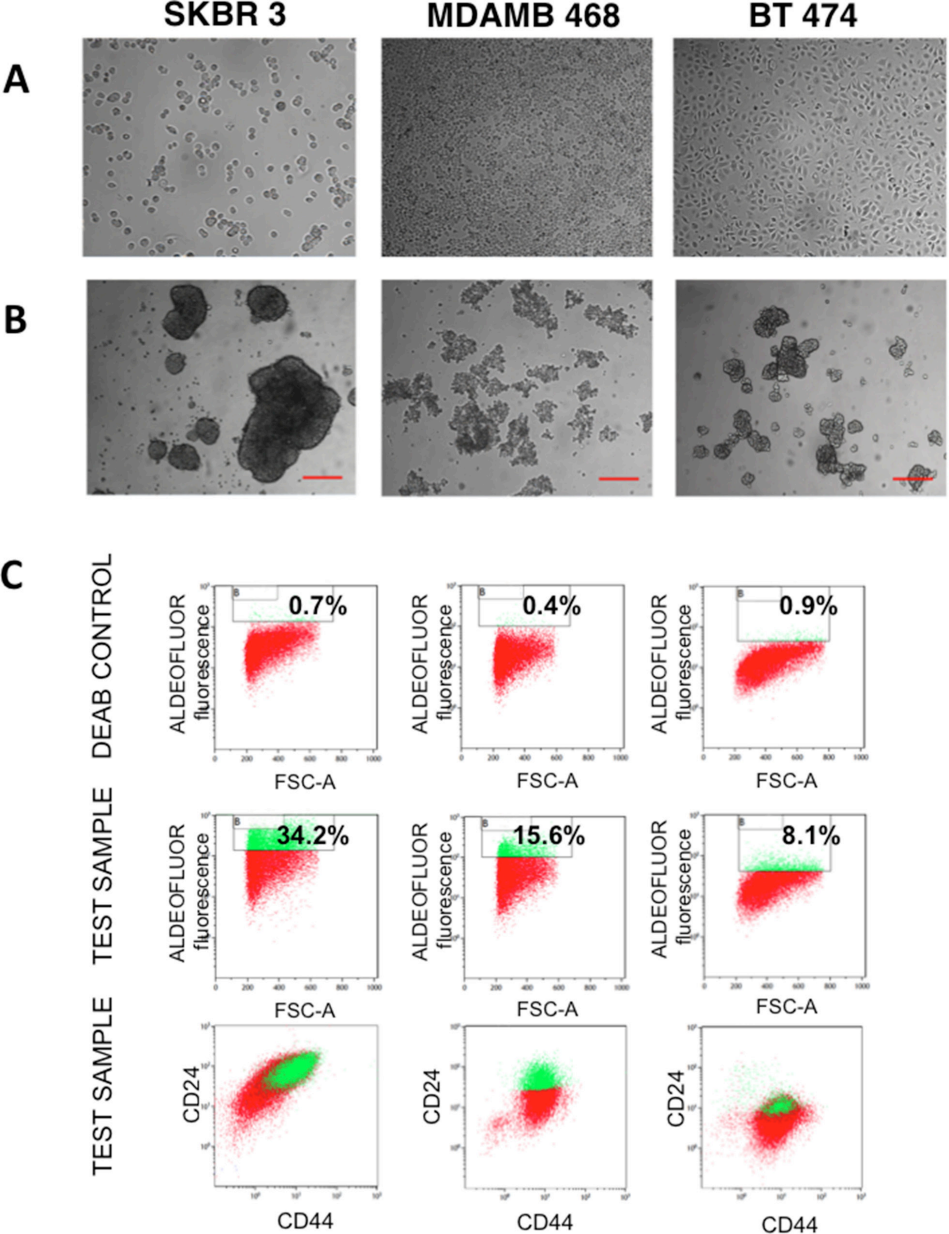
Analysis of breast cancer mammospheres **A.** Phase contrast images of SKBR 3, MDAMB 468 and BT 474 cells grown on the surface of a culture dish. **B.** Phase contrast images of SKBR 3, MDAMB 468 and BT 474 grown in a suspension in the form of mammospheres. Scale bars represent 250 μm. **C.** Flow cytometric assessment of cancer stem-like cells populations in cultures of SKBR 3, MDAMB 468 and BT 474 cells by the combination of stem cell markers ALDH1, CD24 and CD44. First row: cells treated with DEAB, an inhibitor of ALDH1; second row: cells without inhibitor treatment; third row: cells co-labeled for surface antigens CD24 and CD44. Numbers refer to the percentage of cancer stem–like cells.

SKBR 3, MDAMB 468 and BT 474 breast cancer cells were cultured in suspension on a non-adherent substrate in a serum-free medium. After 7 days in culture, mammospheres (Fig. 1A, 1B) were observed for the three cell lines and remained steady for a further 5 days until the culture was terminated. Additionally, mammospheres derived from each cell line showed different morphology. Next, we focused on enrichment of the mammosphere-forming cells for CSC based on common markers including the enzyme aldehyde dehydrogenase (ALDH)^high^ activity and surface expression of CD44 and CD24 [33-36]. Mammospheres enriched for CSC were selected by flow cytometry on the basis of ALDH activity (Aldefluor reagent fluorescence). The cells were co-stained with anti-CD24 and anti-CD44 antibodies. After 7 days in culture, on the average ∼ 10–30% of SKBR 3 and MDAMB 468 cells were Aldefluor positive, whereas only ∼5–10% of BT 474 cells were Aldefluor positive. The stem cell populations sorted from SKBR 3, MDAMB 468 and BT 474 cell lines were enriched in cells with an ALDH^high^ CD44^+^CD24^+^, ALDH^high^ CD44^+^CD24^+^ or ALDH^high^ CD44^+^ CD24^low^ phenotype, respectively (Fig. 1C).

To determine the ability of ALDH^high^ cells to proliferate and form colonies *in vitro*, the 3D-soft agar colony formation assay was performed in SKBR3 ALDH^high^ mammospheres. The plating efficiency (number of colonies) of SKBR3 ALDH^high^ cells was higher and the colony size was larger than for SKBR3 ALDH^low^ cells. The proportion of colonies from three independent experiments was 38.3±9 in SKBR3 ALDH^high^ cells and 12±8 in SKBR3 ALDH^low^ cells (supplementary Fig. 1).

### Induced pluripotent stem cells generated from primary human fibroblasts show rapid proliferation

**Figure 2:**
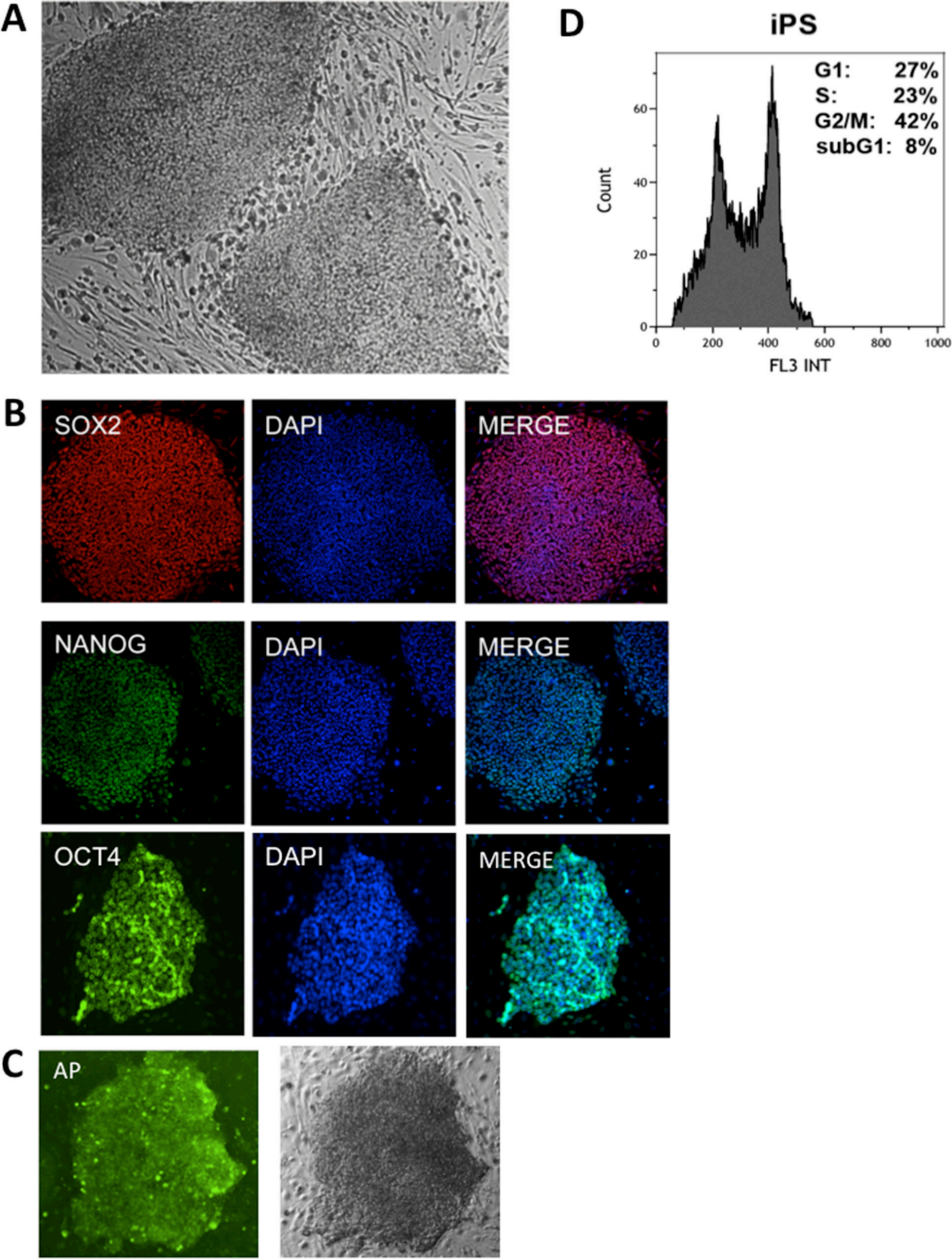
Characterization of human iPS **A.** Human iPS cells observed in phase contrast. **B.** Human iPS labeled with pluripotency markers: anti-SOX2, anti-NANOG and anti-OCT4 with DAPI counterstaining (right). **C.** iPS cells labeled for alkaline phosphatase (AP) and observed in phase contrast. **D.** An example of typical DNA content of iPS cells after propidium iodide labeling measured by flow cytometry.

Primary human fibroblasts were reprogrammed retrovirally and 12 days after transduction, the first iPS colonies were observed (Fig. 2A). To enhance the efficiency of reprogramming, transduced fibroblasts were cultured in hypoxic conditions (5% O_2_) until the first colonies of iPS appeared [37], followed by culturing in normoxic conditions (21% O_2_). To ensure that iPS used in further analysis did not undergo a differentiation process, they were analyzed for the presence of pluripotency markers such as Sox2, Nanog, Oct4 (Fig. 2B) and alkaline phosphatase (AP) (Fig. 2C). Additionally, the cell cycle distribution of the generated iPS cells was examined, showing rapid proliferation with the majority of the cells in S and G2 phases (Fig. 2D).

### The levels of PFKFB3 and PFK1 proteins do not change during the cell cycle in synchronized breast cancer cells

**Figure 3:**
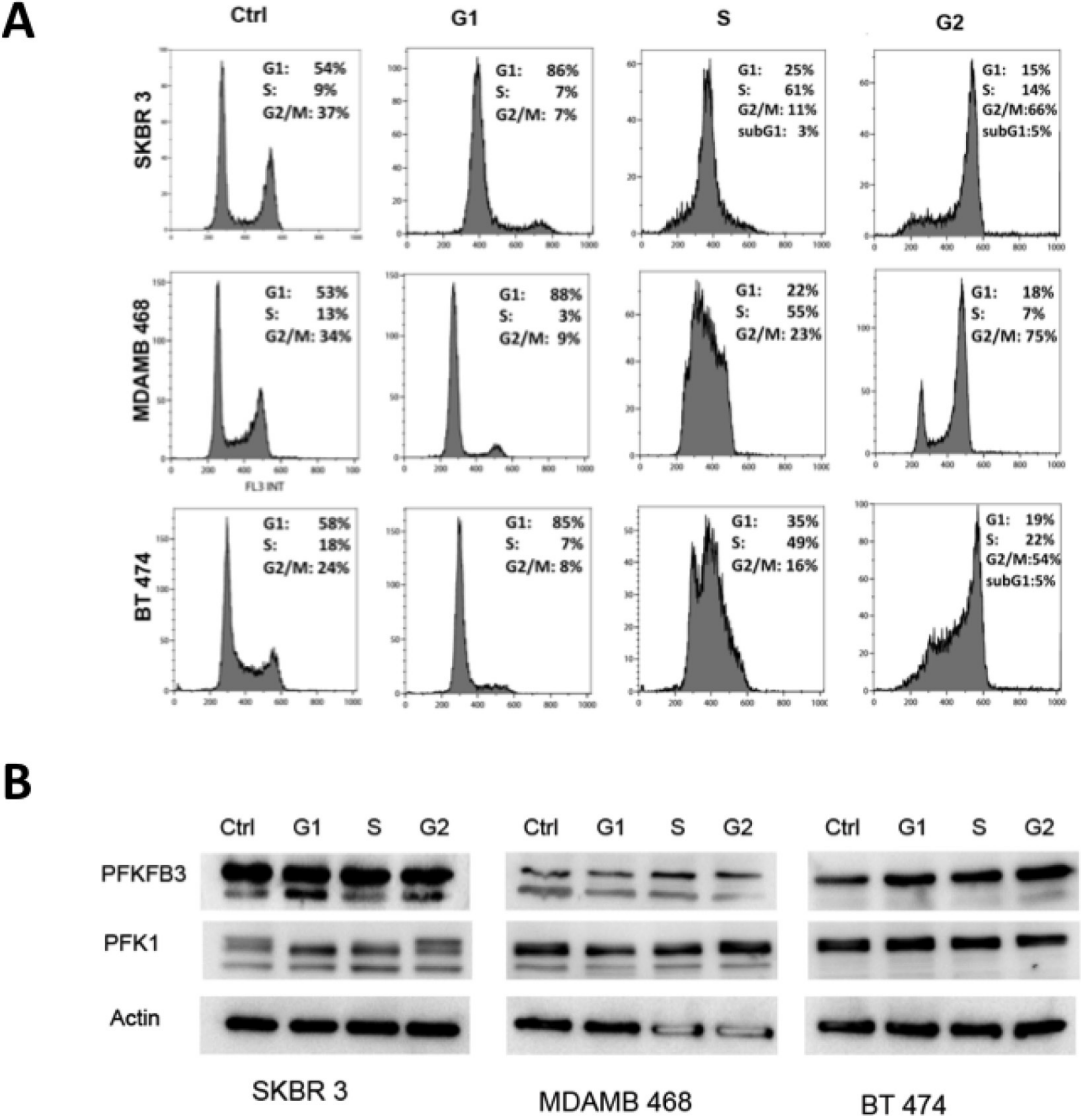

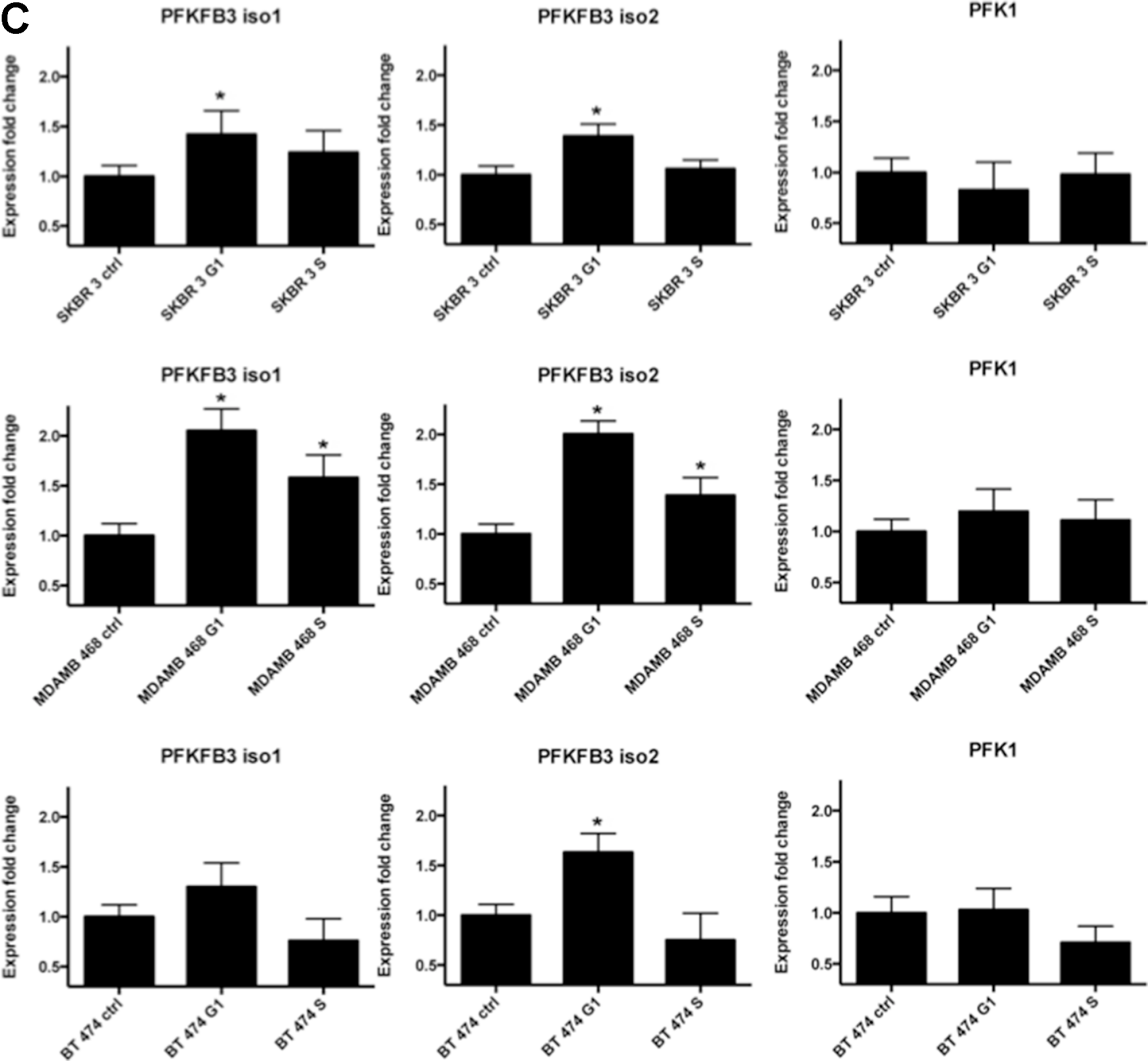
PFKFB3 and PKF1 expression in synchronized breast cancer cells **A.** Cell cycle profiles for SKBR 3, MDAMB 468 and BT 474 cells at different times after DTB and/or nocodazole release as determined by flow cytometry analysis of DNA content. Representative histograms were chosen from several independent experiments. Columns represent respectively unsynchronized (ctrl) cells, cells synchronized in G1 phase, S phase and G2 phase. **B.** Western blot analysis using anti-PFKFB3 and anti-PFK1 antibodies of proteins from asynchronized (Ctrl) and synchronized (G1,S, G2) SKBR 3, MDAMB 468 and BT 474 cells. **C.** Relative expression of *PFKFB3* (both isoforms) and *PFK1* genes in SKBR3, MDAMB 468 and BT 474 cells (ctrl, asynchronized; G1, synchronized in G1 phase; S, synchronized in S phase). Fold change (compared to asynchronized cells) was calculated from the ΔΔCT values by the formula 2^−ΔΔCT^ and the data are represented as the mean ± SD from triplicate measurements and 3–4 independent experiments. *Represents statistically significant increase in gene expression (*P* < 0.05).

To test the hypothesis that PFKFB3 and PFK1 expression is regulated in a cell cycle-dependent manner, the level of PFKFB3 protein was analyzed in breast cancer cells (SKBR 3, MDAMB 468, BT 474) synchronized using a double thymidine block (DTB) and/or nocodazole treatment. Progression through the cell cycle was monitored by the release of cells from DTB at selected time points (Fig. 3A), using unsynchronized cancer cells as controls. No significant changes in PFKFB3 and PFK1 protein expression at various stages of the cell cycle were observed (Fig. 3B). Interestingly, these cell lines differed in the expression of different isoforms of PFKFB3 and PFK1 (m-muscle, l-liver, p-platelet). SKBR 3 and MDAMB 468 cells expressed both isoforms of PFKFB3 and three isoforms of PFK1, whereas BT 474 cells expressed only one isoform of PFKFB3 and PFK1 (Fig. 3B).

Next, SKBR 3, MDAMB 468 and BT 474 cell extracts were analyzed by qRT-PCR. Our results showed an increased level of PFKFB3 mRNA during the G_1_ phase of the cell cycle. However, the PFK1 mRNA level in synchronized cancer cells remained unchanged throughout the cell cycle (Fig. 3C).

### PFKFB3 and PFK1 protein expression is lower in iPS cells than in cancer and CSC

**Figure 4:**
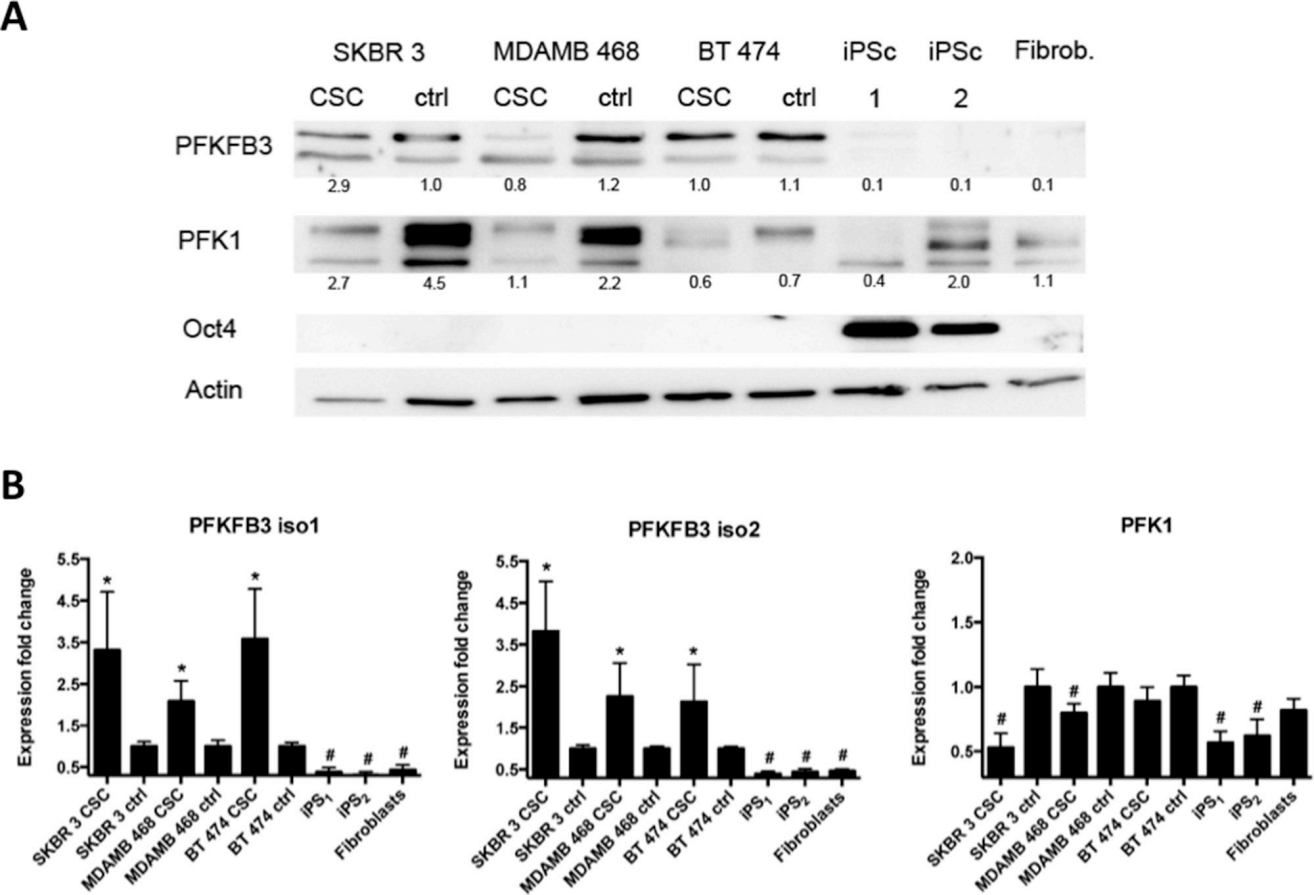
Endogenous PFKFB3 and PKF1 expression in asynchronized cancer cells, CSC and iPS cells **A.** Western blot analysis of: cancer (ctrl); cancer stem cells (csc) from SKBR 3, MDAMB 468 and BT 474 cells; iPS cells and fibroblasts. Cell lysates were examined for the presence of PFKFB3, PFK1, and Oct-4. “iPS 1” shows iPS cells from the RIKEN cell bank, whereas “iPS 2” represents cells obtained by reprogramming in our laboratory; numbers represent values from densitometric quantification. Values represent relative signals normalized to β-actin. **B.** Relative expression of *PFKFB3* and *PFK1* genes in SKBR 3, MDAMB 468 and BT 474 cancer cells, in the corresponding cancer stem cells, iPS cells and fibroblasts. Fold change (compared to unsynchronized cancer cells- denoted as “ctrl”) was calculated from the ΔΔCT values with the formula (2^−ΔΔCT^) and the data are represented as the mean ± SD from triplicate measurements from three independent experiments. *Represents a statistically significant increase in gene expression, whereas ^#^ represents a statistically significant decrease in gene expression (*P* < 0.05).

We next analyzed the expression profile of PFKFB3 and PFK1 proteins in FACS-sorted breast cancer cell line-derived CSC, iPS cells, and human primary fibroblasts (the material for production of iPS cells) by Western blot and qRT-PCR. Western blot analysis showed that PFKFB3 is undetectable in iPS cells and human primary fibroblasts when compared with cancer and CSC. Moreover, PFKFB3 expression in cancer cells and CSCs is comparable. The pluripotency of iPS cells during the experiment was controlled by Oct4 expression. Relatively low expression of PFK1 was observed in both CS- and iPS cells, in contrast to its high expression in cancer cells (Fig. 4A).

Similar results were obtained by qRT-PCR; the level of PFK1 and PFKFB3 mRNA in iPS cells was significantly decreased in comparison to that in CSC cells. In comparison to the Western blot analysis, significantly higher expression of PFKFB3 mRNA was observed in CSC than in differentiated cancer cells. The increased level of PFK1 mRNA coincided with higher expression of PFK1 in cancer cells when compared to cancer cell-derived mammospheres (Fig. 4B).

### Higher PFKFB 3 mRNA expression in CSC is accompanied by down-regulation of PFK1

As shown in Fig. 4B, significant upregulation of the PFKFB3 mRNA level was observed in all three breast cancer cell line-derived CSC compared with the parent breast cancer cells. Additionally, we observed that increased expression of PFKFB3 mRNA was accompanied by downregulation of PFK1 mRNA in CSC (Fig. 4B). Similar results were obtained after densitometric quantification of PFK1 expression in cancer- and CS cells (Fig. 4A).

### Hypoxia induces PFKFB3 and decreases PFK1 expression in cancer-, CS- and iPS cells

**Figure 5:**
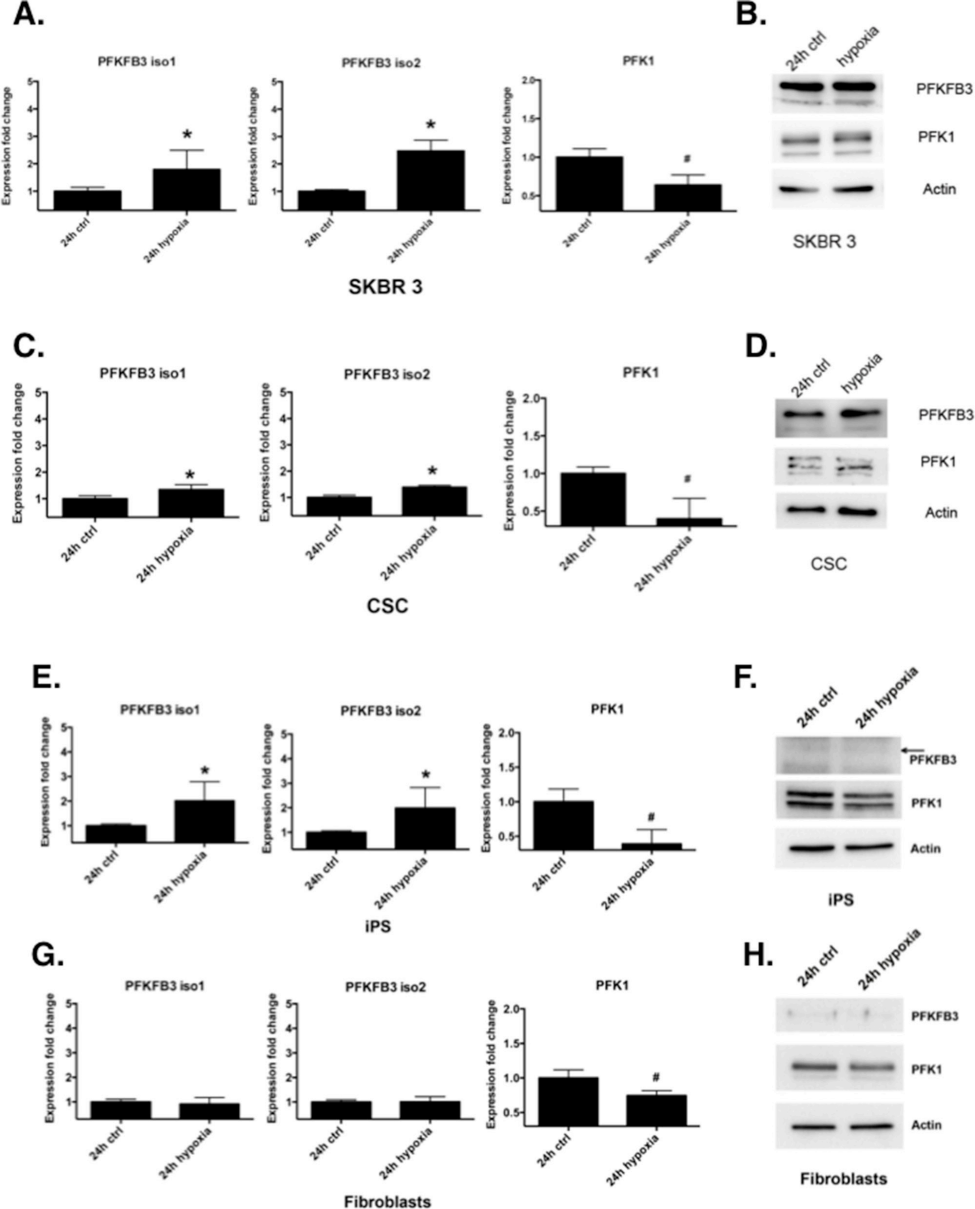
Regulation of PFKFB3 and PFK1 by hypoxia **A, C, E, G.** Relative level of *PFKFB3* and *PFK1* mRNA in SKBR 3 cells, CSC, iPS cells and primary dermal fibroblasts cultured for 24 h in normoxic conditions (ctrl) or in 5% hypoxia, respectively. Fold change (compared to cells cultured in 20% oxygen level) was calculated from the ΔΔCT values with the formula (2^−ΔΔCT^) and the data are represented as the mean ± SD from triplicate measurements from three independent experiments. *Represents a statistically significant increase in level, whereas ^#^ represents a statistically significant decrease (*P* < 0.05). **B, D, F, H.** Western blot analysis of SKBR 3 cells, CSC, iPS cells and primary dermal fibroblasts cultured for 24 h in normoxic conditions (ctrl) or in 5% hypoxia.

PFKFB3 possess a hypoxia-responsive element, which leads to induction of PFKFB3 in various cancer cell lines [38]. We examined the effect of hypoxia on PFKFB3 and PFK1 gene and protein expression in cancer-, CS-, iPS cells and fibroblasts. Gene expression analysis showed that the PFKFB3 mRNA level is several fold higher in cancer, CSC and iPS cells cultured in hypoxic conditions compared to normoxic conditions. At the same time, the PFK1 mRNA level was significantly downregulated upon hypoxia (Fig. 5A, 5C, 5E). Similar results were obtained by Western blot analysis (Fig. 5B, 5D, 5F). The PFKFB3 mRNA level in fibroblasts cultured under hypoxic conditions remained unchanged, whereas the PFK1 mRNA level was significantly decreased compared to that in normoxic conditions (Fig. 5G, 5H).

### PFKFB3 expression is downregulated upon inhibition of glycolysis

**Figure 6:**
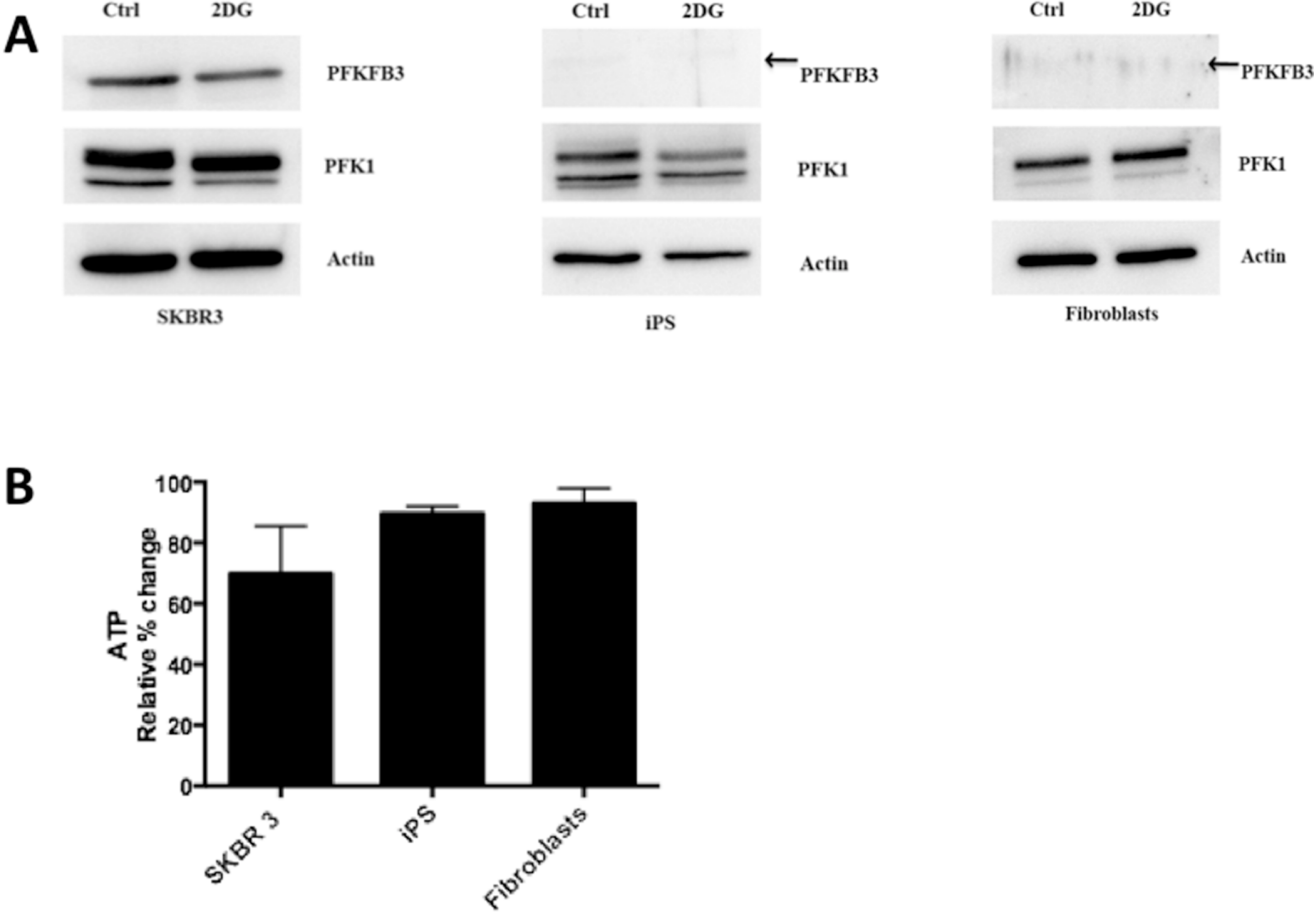
Inhibition of glycolysis by 2-DG **A.** Western blot for PFKFB3 and PFK1 expression in untreated cells (Ctrl) and cells growing with 2-DG. **B.** Relative % changes in the ATP level upon inhibition of glycolysis in SKBR 3 cells, fibroblasts, and iPS cells.

As PFKFB3 plays an important role in glycolysis, we examined the effect of inhibiting glycolysis on PFKFB3 expression and ATP level in cancer-, iPS cells, and fibroblasts. Expression of PFKFB3 in SKBR3 breast cancer cells decreased after 24 h incubation with 10 mM 2-deoxy-D-glucose (2-DG) (Fig. 6A). The low endogenous expression level of PFKFB3 in untreated iPS cells and fibroblasts hampered drawing conclusions whether inhibition of glycolysis had an impact on PFKFB3 expression in these cells. Inhibition of glycolysis by 2DG caused ∼30% reduction of ATP in SKBR3 cells, whereas in iPS cells and fibroblasts inhibition of glycolysis had a weaker impact on the cellular energy pool (Fig. 6B). We could not measure ATP in CSC upon inhibition of glycolysis, since the procedure for sorting them (staining with fluorescence markers) prevented using luminescent ATP detection kits.

### PFKFB3 knockdown suppresses glycolysis in cancer and iPS cells

**Figure 7:**
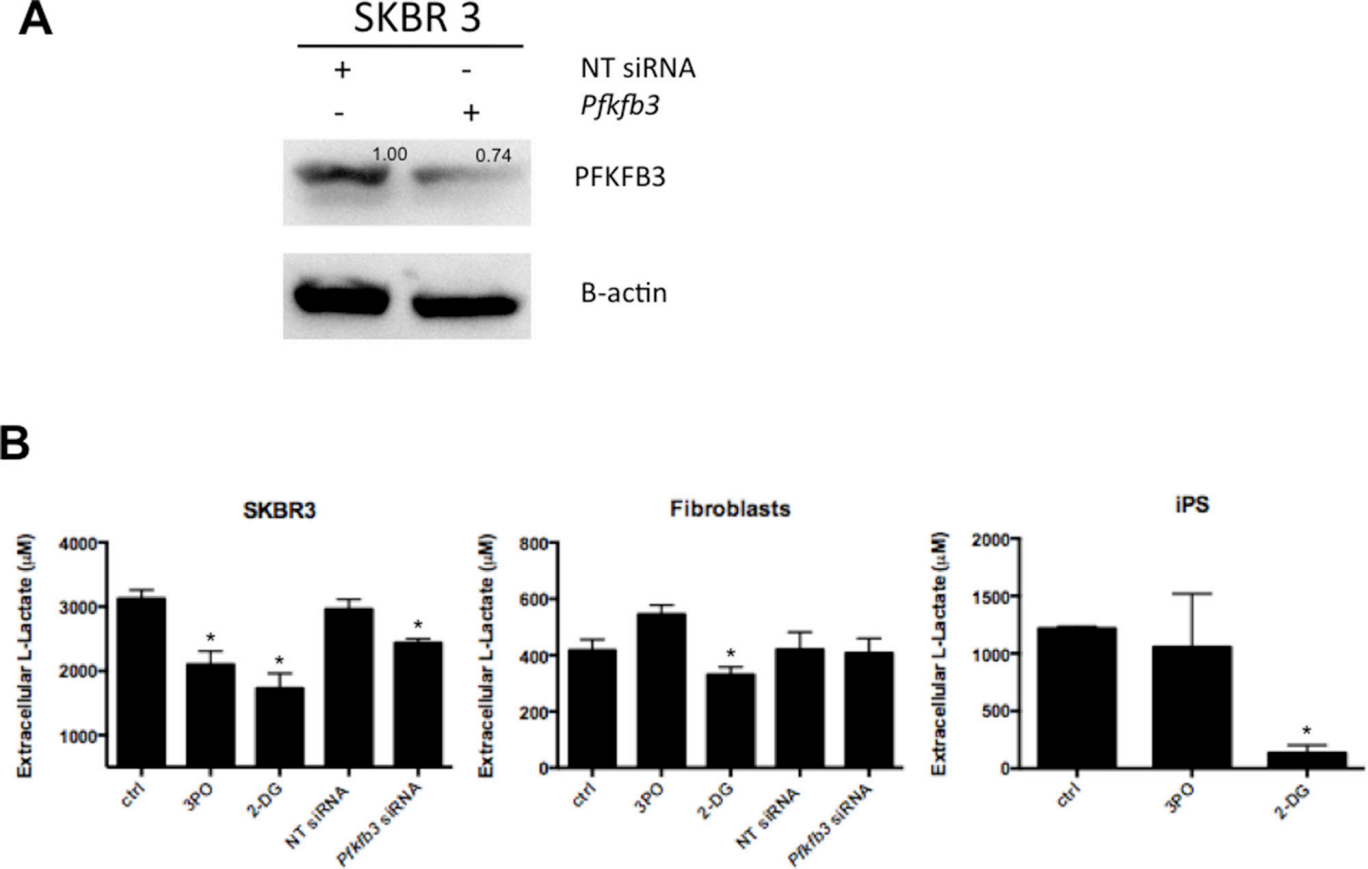
Effect of PFKFB3 knockdown on lactate production **A.** Western blot for PFKFB3 expression in SKBR 3 cells transfected with PFKFB3 siRNA or nontargeting (NT) control siRNA; numbers represent values from densitometric quantification. Results were normalized by arbitrarily setting the density of the control sample to 1.0. **B.** SKBR 3 cells, fibroblasts and iPS cells were treated with 3PO, 2DG or transfected with either NT siRNA or PFKFB3 siRNA and cultured for 24 h. Lactate was determined in conditioned media by a fluorescence-based method. Results were compared with a standard curve and expressed as a total concentration (nM) of extracellular lactate produced in 24 h/cell; *Represents a statistically significant decrease of lactate generation (*P* < 0.05).

We next assessed the effect of PFKFB3 inhibition on glycolytic activity by measuring the lactate production. Initial validation using PFKFB3 siRNA showed ∼30% of silencing in all breast cancer cell lines analyzed. The SKBR 3 breast cancer cell line was selected to conduct further analysis upon PFKFB3 silencing (Fig. 7A). Additionally, the effect of the PFKFB3 inhibitor, 3-(3-pyridinyl)-1-(4-pyridinyl)-2-propen-1-one (3PO) and 2-DG on lactate production was tested.

Selective targeting of PFKFB3 by siRNA and 3PO resulted in a significant decrease of extracellular lactate in SKBR 3 cells. A moderate effect of 3PO on lactate level in iPS cells was also observed. The level of extracellular lactate in fibroblasts was not affected upon 3PO treatment or siRNA silencing of PFKFB3. A more aggressive inhibition of glycolysis with 2DG potently reduced the level of extracellular lactate in all three cellular systems (Fig. 7B).

### Inhibition of PFKFB3 by 3PO induces G2 cell cycle arrest in cancer cells

**Figure 8:**
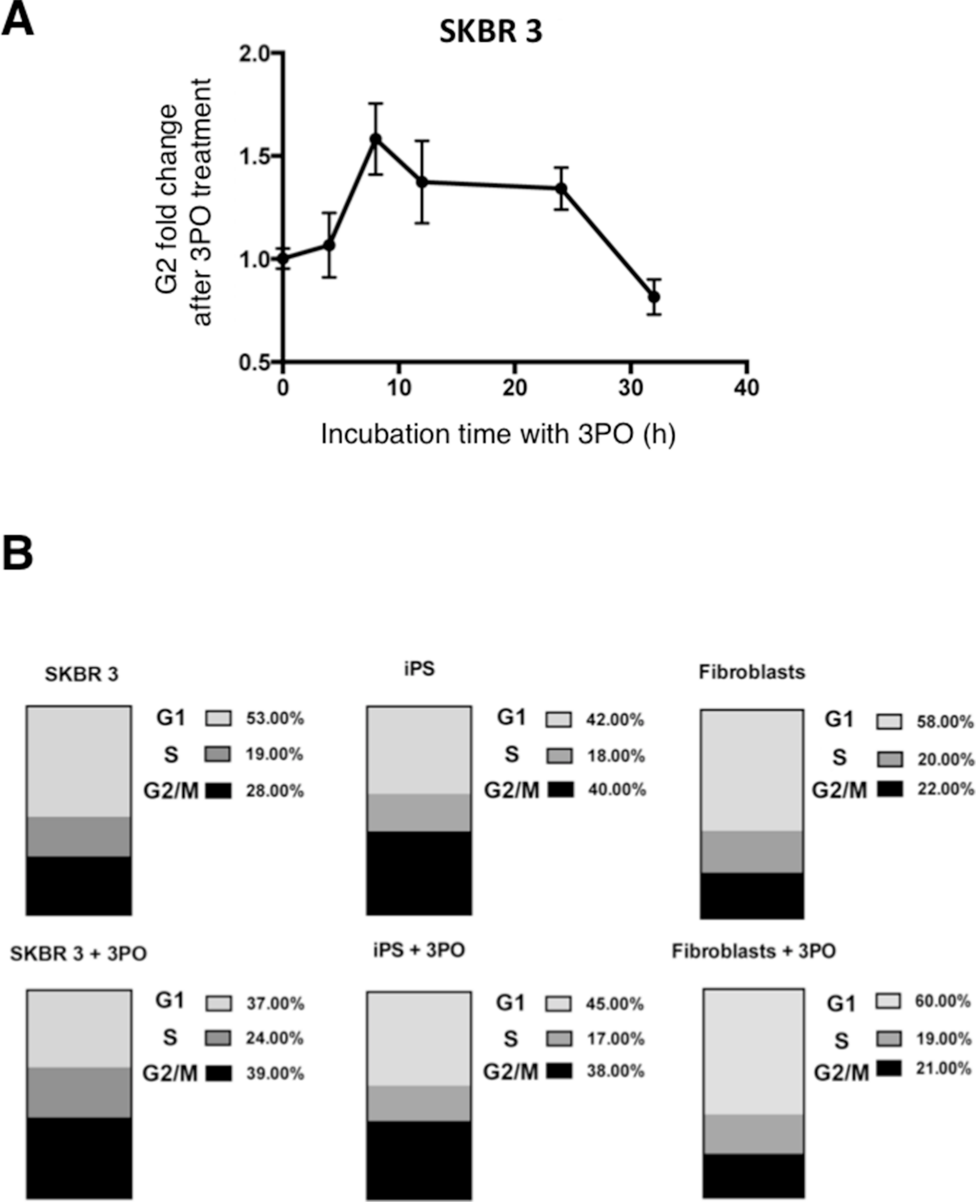
Effect of 3PO on cell cycle phase distribution **A.** Changes of the G_2_ fraction of SKBR 3 cells following addition of the PFKFB3 inhibitor 3PO (10 μM). Data represent mean values from four independent experiments +/− SD. **B.** Effect of PFKFB3 inhibition on the distribution of DNA content in SKBR 3 cells, fibroblasts and iPS cells. Upper panel, untreated cells; lower panel, cells treated with 10 μM 3PO.

To examine if PFKFB3 has a significant influence on cell cycle phase distribution in cancer-, iPS cells and fibroblasts, the effect of 3PO was studied. The presence of the PFKFB3 inhibitor triggered cell cycle arrest of SKBR 3 cells in the G2 phase, and the most marked effect was observed after 8–12 h incubation (Fig. 8A). In contrast to Clem's [39] experiments, the arrest of cells in the G2 phase was temporary, suggesting that after ∼24 h the cells could overcome the effect of PFKFB3 inhibition and divide further. Enrichment by more than 10% of cells in the G2 phase after growth for 12 h with 3PO was also observed in SKBR 3 cells (Fig 8B). Fibroblasts and iPS cells showed an increase of 7% and 3% in cells trapped in the G1 phase upon 3PO treatment, respectively (Fig. 8B).

## DISCUSSION

Metabolic regulation is tightly coupled to cell proliferation and tumorigenesis, and signaling by metabolic enzymes influences cell cycle transitions in order to determine cell fate. iPS-derived tissues have a high propensity for cancer development, and the metabolic state of fast-proliferating iPS cells and tumor-initiating CSCs might contribute to their final phenotype and function. Thus it is likely that glycolysis-promoting enzymes like PFKFB3 may be differentially regulated in iPS, CSCs and cancer cells.

In this work we studied two glycolysis-related enzymes, PFKFB3 and PFK1, in distinct cellular systems (iPSs, cancer cells, CSCs and fibroblasts) in order to capture differences in cell cycle regulation and metabolic activity. Three breast cancer cell lines (SKBR 3, MDAMB 468, BT 474), breast cancer cell line-derived CSCs, iPS cells from two independent sources, and human dermal fibroblasts were chosen for analysis. In contrast to previous reports [20], we did not observe cell cycle-dependent changes in PFKFB3 expression in synchronized breast cancer cells, a discrepancy which could result from possible overexpression of PFKFB3 in the cell lines analyzed. High expression of PFKFB3 was observed in various types of cancer including colon, prostate, lung, breast, pancreas, thyroid, and ovarian tumors as well as in leukemias [29, 39-41]. A relatively high expression of PFKFB3 was also reported in asynchronous breast cancer cell lines and HeLa cells, which shows that cell cycle synchronization is not always required for the detection of PFKFB3 [20, 28].

However, synchronization of SKBR 3, MDAMB 468 and BT 474 cells resulted in a significant elevation of PFKFB3 mRNA level during the G1 phase as compared to asynchronized cells. An earlier report demonstrated that elevated expression of the *PFKFB3* gene precedes the appearance of the protein by ∼2 h, and that its maximum level can be observed for another 2 h [20]. A detailed kinetic study of *PFKFB3* gene expression was not conducted during our analyses, but measurement of the expression of PFKFB3 mRNA at only one time point (12 h) after releasing cells from DTB was enough to observe elevated *PFKFB3* gene expression in cells synchronized in the G1 phase.

Other studies have also reported an elevated level of PFKFB3 in proliferating cells [42, 43], which encouraged us to comparatively investigate its expression level in other fast-dividing cells such as CSCs and iPSs. Unlike CSCs and their parental cells, iPS cells exhibited a very low level of PFKFB3 expression. It has been shown that the unique proliferative properties of human ES cells are partially due to the lack of a growth factor-dependent R point [44]. Since ES cells share important properties of self-renewal and pluripotency with iPS cells, it is likely that they possess similar regulation of the cell cycle. On the other hand, expression of PFK1 (at both the mRNA and protein levels) in iPS cells was comparable to that in CSCs, which indicates that the activation of PFK1 in iPS cells may be PFKFB3-independent.

Furthermore, the analyses of *PFKFB3* and *PFK1* mRNA expression present an interesting pattern in cancer cells and cancer cell-derived CSCs. CSCs revealed a several-fold increase in *PFKFB3* expression compared to cancer cells, which was correlated with downregulation of *PFK1*. It is interesting that PFKFB3 expression in all three types of breast CSCs is comparable, regardless of the heterogenous nature of breast cancer, which is indicated here by differential expression of CD24 [45]. The differences in PKFB3 and PFK1 expression profile in cancer and CSCs seem to be associated with the stem-like and differentiated phenotype of cancer cells, and comprise a way to distinguish cancer cells from CSCs. Since PFKFB3 is an allosteric activator of PFK1, we would expect that an elevated PFKFB3 level would be accompanied by an increase of *PFK1* gene expression. The observed downregulation of *PFK1* may reflect a negative feedback mechanism already reported by others. It has been shown by Telang and colleagues [46] that in some cells the relationship between PFKFB3 expression and intracellular F2,6BP levels may not be so straightforward; elevated PFKFB3 expression was accompanied by a decrease in intracellular F2,6BP and F2,6BP levels were also not directly correlated with glycolytic flux, and it was hypothesized that this could result from elevated glycolysis leading to a negative feedback compensation or from increased use of F2,6BP as a glycolytic substrate for PFK1 [47]. In contrast to mRNA expression data, elevated expression of PFKFB3 was detected only in SKBR 3-derived CSCs by Western blots, suggesting increased degradation or differential regulation of *PFKFB3* gene expression in CSCs sorted from MDAMB 468 cells or BT 474 breast cancer cells.

Another interesting issue associated with glucose metabolism and cancer cell proliferation is the influence of hypoxia on PFKFB3 and PFK1 expression. It is well established that glycolysis is upregulated to enhance energy production under a reduced level of oxygen, and an elevated glycolytic flux is crucial especially for tumor cells exposed to a hypoxic microenvironment [48]. Besides the elevated glycolytic flux, cancer cells produce high levels of lactate and pyruvate as well as showing increased expression of glycolytic enzymes and glucose transporters *via* a HIF-dependent mechanism [49, 50]. Our results showed that hypoxia increased the expression of PFKFB3 in cancer and iPS cells, which is in agreement with other reports. Bobarykina and colleagues [50] showed that the *PFKFB3* gene was expressed in gastric and pancreatic cancer cells and responded strongly to hypoxia via an HIF-1α dependent mechanism. As in our work, these authors could not find a correlation between PFKFB3 mRNA level and protein expression in the cells examined, either in normoxic or hypoxic conditions.

Our data demonstrate also that the exposure of cancer cells to hypoxia changed the expression of PFKFB3 and PFK1 to levels resembling those in CSCs. It has been shown by other groups that hypoxia can induce stem cell-like transcription phenotypes in myeloma and glioblastoma cells as well as enhance CSC proliferation [51-53]. It seems that CSCs have enhanced glycolysis due to hypoxia-mediated modulation of R-point markers such as PFK1 and PFKFB3.

*PFKFB3* gene silencing has been shown to decrease glycolysis, induce cell-cycle delay, and inhibit anchorage-independent growth in HeLa cells [29]. Inhibition of PFKFB3 by the specific inhibitor, 3PO decreases F2,6BP levels, which in turn decreases PFK1 activity and suppresses glycolytic flux with a cytostatic effect [39]. Furthermore, the exposure of cells to 3PO has been shown to attenuate deoxy-D-glucose uptake, lactate secretion, TNF-α secretion, T cell aggregation, and proliferation, and 3PO displays significant immunosuppressive activity *in vivo* [54]. In our study, both pharmacological and genetic knockdown of PFKFB3 expression influenced the glycolytic activity in cancer cells and iPS cells. In addition, inhibition of glycolysis by 2DG resulted in reduction of PKFB3 expression as well as decrease of extracellular lactate. Inhibition of glycolysis was also associated with ATP depletion, however the degree of ATP reduction in fibroblasts and iPS cells might suggest resistance of these cells to inhibition of glycolysis or lower dependence on glycolysis for energy production. Unlike cancer cells and iPS, fibroblasts were unaffected by 3PO treatment, which was similar to a previous report showing that transformed fibroblasts (PFKFB3^+/−^) are more sensitive to this inhibitor than wild-type control fibroblasts (PFKFB3^+/+^) [39].

Furthermore, inhibition of PFKFB3 by 3PO resulted in accumulation of cells in the G_2_ phase of the cell cycle in breast cancer cells, but not in iPS cells and fibroblasts. This phenomenon has been already observed after 3PO and rhodamine-123 treatment [39, 55] suggesting the existence of energetic checkpoints, which prevent incompetent cell division. The G2-M energetic checkpoint at which the SKBR 3 cells were trapped is likely to be ATP-dependent and a 30–40% reduction of whole-cell ATP is required for this accumulation [55]. Nevertheless, we observed a minor increase of the G1 population after 3PO treatment in iPS cells, which together with a moderate, 3PO-mediated lactate reduction does not exclude the hypothesis that cell cycle regulation in these cells is PFKFB3-dependent.

In summary, we have demonstrated that two glycolysis-promoting, R-point-specific enzymes, PFKFB3 and PFK1, may serve as tool enabling discrimination of CSCs from non-stem cancer cells as well as iPS cells. Our results provide a new insight into the molecular mechanism of PFKFB3-regulated cell cycle progression and arouse hope for developing more effective anti-cancer therapies. However, implementing this proof-of-concept in primary cancer cells is advisable to strengthen our conclusions.

## MATERIALS AND METHODS

### Cell culture

Breast cancer cells: SKBR 3, MDAMB 468 and BT 474 were cultivated in RPMI-1640 medium (PAA, Pasching, Austria) supplemented with 10% v/v inactivated fetal bovine serum (FBS) (PAA, Pasching, Austria) and 1% v/v penicillin streptomycin (PS) (Gibco, USA). Cells were maintained at 37°C in a humidified atmosphere with 5% CO_2_ and passaged upon confluency (70%–80%) using a trypsin-EDTA solution (PAA, Pasching, Austria) and suspended into fresh medium. Primary human fibroblasts, provided by Dr. G. Kratz [56], were cultivated in DMEM High Glucose medium (PAA, Pasching, Austria) supplemented with 10% v/v FBS (PAA) and 1% v/v PS (Gibco).

### Mammosphere generation from breast cancer cell lines

Cultures of SKBR 3, MDAMB 468 and BT 474 cells at ∼70% confluence were trypsinised as described above and transferred into non-adherent T-175 flasks (Sarstedt) coated with 20 mg/ml poly-2-hydroxyethyl methacrylate (polyHEMA) (Sigma). Cells were cultivated in serum-free Mammary Epithelial Cells Media (Promocell) supplemented with Bovine Pituitary Epithelial (BPE) (0.004 mg/ml), Recombinant human Epidermal Growth Factor (hEGF) (10 ng/ml), recombinant human insulin (5 μg/ml), and hydrocortisone (0.5 μg/ml) (Promocell). Cells were maintained at 37°C with 5% CO_2_ for at least 5 days after the appearance of mammospheres.

### Retrovirus production and generation of iPS cells

Induced pluripotent stem (iPS) cells were generated from primary human dermal fibroblasts by overexpressing a set of transcription factors called reprogramming factors. Four reprogramming factors were chosen: Oct4, Sox2, Klf4 and c-Myc, and were transduced into the target cells using retroviral vectors. Briefly, retroviruses containing genes for the 4 factors were produced independently by transfecting HEK 293 cells (X-tremeGENE HP DNA Transfection Reagent, Roche Diagnostics) with vectors encoding human Oct4, Sox2, Klf4, c-myc and GFP, the packaging vector pUMVC, and the envelope plasmid pCMV-VSV-G (all plasmids were kindly provided by Dr. Oliver Rothfuss). Plates with transfected cells were incubated at 37°C with 5% CO_2_ and viral supernatants were collected after 24, 48, 72 h post-transfection and stored at 4°C. The day before viral transduction, 6-well plates containing 50,000 human dermal fibroblast cells/well) in complete DMEM medium supplemented with 10% FBS and 1% PS were prepared. Next the human dermal fibroblasts were transduced with the previously collected viral supernatants (MOI of 10) and incubated at 37°C with 5% CO_2_ for 6 days and the medium was changed every second day. Six days after transduction the cells were treated with acutase (15 min), seeded on a MEF-feeder- layer (provided by Dr. Oliver Rothfuss) with Primate ES Cell Medium (ReproCELL) supplemented with 1 mM valproic acid and 10 μM ROCK inhibitor and cultured in hypoxic conditions (5% O_2_) in order to enhance the reprogramming efficiency. After the first colonies were formed on the plate (between 10–12 days) iPS cells were transferred into normoxic conditions (21% O_2_). In parallel, human iPS cells (RBC-HPS0063, cell line name: 201B7) from the RIKEN BioREsource Center (Japan) were cultured under the same conditions as the iPS cells obtained after viral transduction.

### Immunocytochemical analysis of human iPS cells

Human iPS cells were fixed in 4% paraformaldehyde (Santa Cruz Technology) for 20 min at room temperature (RT), washed 3 × 5 min in PBS, and permeabilized with 0.2% Triton X-100 (Sigma) for 15 min at RT. Blocking was performed by incubation in 1% BSA/PBS (1 h, RT). Cells were incubated overnight at 4°C with primary antibodies directed against Nanog (Stemgent), Sox2 (R&D systems) or Oct4 (Stemgent). After washing (3 × 5 min in PBS) the cells were labeled with respective secondary antibodies conjugated with a fluorophore for 1 h at RT. After washing (3 × 5 min in PBS) cells were mounted in Vectashield® mounting medium with DAPI (Vector Laboratories, Inc., USA). Alkaline phosphatase (AP) staining was performed using fluorescent Alkaline Phosphatase Live Stain kit (Life Technologies) according to the manufacturer's recommendations. Fluorescence images were captured using a Zeiss inverted LSM700 confocal microscope (Carl Zeiss GmbH, Germany).

### Aldefluor assay

The ALDEFLUOR™ kit (StemCell Technologies) was used to isolate the population with a high aldehyde dehydrogenase (ALDH) activity. Briefly, breast cancer cells grown in suspension were peletted by centrifugation (250 × g, 5 min), washed with PBS, trypsinised at 37°C and mammospheres were disaggregated by gentle pipetting. After centrifugation the cells were suspended in ALDEFLUOR™ Assay Buffer containing ALDH substrate (BAAA) at 1 × 10^6^ cells/ml and incubated for 45 min at 37°C. Next, the cells were re-suspended in ALDEFLUOR™ Assay Buffer and placed on ice. As a negative control, for each sample of cells an 0.5 ml aliquot was treated with 5 μl of DEAB, a specific ALDH inhibitor. ALDH activity was measured by fluorescence-activated cell sorting (FACS) analysis on a Gallios flow cytometer (Beckman Coulter, Gallios™, USA) and data were evaluated with Kaluza software (Beckman Coulter).

### FACS analysis

For better stem cell marker profiling, two fluorochrome-conjugated monoclonal antibodies were used: APC mouse anti-human CD 44 (BD Biosciences) and PE mouse anti-human CD 24 (BD Biosciences). Antibodies were added to the cell suspension previously stained with the ALDEFLUOR kit, as recommended by the manufacturer, incubated at 4°C in the dark for 20 min, centrifuged (250 × g, 5 min), and suspended in PBS. Labeled cells were analyzed using the BD FACS Aria III (BD Biosciences). In order to diminish the spectral overlap signal between ALDEFLUOR and CD24-PE, the single-labeled, double-labeled and unlabeled samples were analyzed and data was compensated using Kaluza software. The spectral overlapping signal was subtracted from the total fluorescence detected in every channel.

The sorting gates were established using a negative control of ALDEFLUOR-stained cells incubated with DEAB. Sorted CSC (ALDH^high^) were used directly for Western blotting, qRT-PCR analysis and 3D-soft agar assay.

### Cell cycle profiling

Cells were fixed in cold ethanol (70%) on ice for 1 h, washed with PBS, resuspended in 50 μl PBS, and incubated with 100 μg/ml RNase A (Sigma) at 37°C for 15 min, followed by addition of propidium iodide (PI) (Sigma; 100 μg/ml) at RT for 10 min. DNA content was assessed by a Galios flow cytometer (Beckman Coulter) using at least 20,000 cells per sample and data were analyzed using Kaluza software (Beckman Coulter).

### Soft agar assay

Soft agar assay was performed in a 6-well plate with each well containing two layers of agarose (Calbiochem) in culture medium (RPMI). The lower layer of well consisted of 0.5% agarose while the upper layer contained 0.35% agarose. Sorted ALDH^high^ cells were incubated in suspension overnight, counted and added into the upper layer of agarose. The cells were then incubated for 2–3 weeks and the culture medium was changed twice a week. Colony formation was observed and colonies were stained with 0.005% crystal violet (Acros Organics).

### Cell synchronization

Cancer cells were synchronized using a double thymidine block (DTB) (G1/S block) and subsequently released for several time intervals (0 h-12 h). Cells synchronized in the G1/S phase were collected at the time of thymidine removal from the medium (0 h), whereas cells synchronized in G0/G1 were collected 6–12 h later. Briefly, cells were incubated in RPMI supplemented with 3 mM thymidine (Sigma) for 24 h, washed three times with PBS, incubated in complete medium without thymidine and grown for another 12 h prior to a second incubation with thymidine for 24 h. After release from the second block, cells were grown in complete DMEM followed by harvesting at selected time points. For G2/M phase synchronization cells were treated with 100 ng/ml of nocodazole (Sigma) for 12–20 h prior to harvesting.

Cell cycle stages were determined by FACS analysis and expressed as percentages of G0/G1, S and G2/M cells, respectively. During synchronization of the cell cycle, the PFKFB3 inhibitor 3-(3-pyridinyl)-1-(4-pyridinyl)-2-propen-1-one (3PO) (10 μM) (kindly provided by Amy Clem, University of Louisville, USA) was added to the cells for 0 h-32 h prior to harvesting.

### Lactate determination

Extracellular L-lactate was assessed using the L-lactate Assay kit (Cayman Chemical) according to manufacturer's recommendations. Briefly, 24 h before sample collection, cell culture medium was replaced with a fresh one and appropriate drugs (3PO, 2DG) were added. Cell-free supernatant was collected, mixed with lactate kit reagents and incubated for 20 min at room temperature as indicated by manufacturers. At the same time number of cells was counted. Fluorescence was read using an excitation wavelength of 530–540 nm and emission wavelength of 585–595 nm. Results were compared with standard curve and expressed as a total concentration of extracellular lactate produced in 24 h/ cell.

### RNA interference

Cells were plated at a density of 200,000 cells/well in a 6-well plate in 2.5 ml complete medium and 24 hours after seeding were transfected with either 20 nM PFKFB3 siRNA (HSS107860, Life Technologies) or control siRNA (Stealth RNA siRNA Negative Control Medium GC Duplex) (Life Technologies). Transfection was carried out in serum-free and antibiotic-free medium using X-tremeGENE HP DNA Transfection Reagent (Roche Diagnostics) following the manufacturer's instructions. Cells were incubated in complete medium at 37°C for 48 hours before cell harvest or further experiments. Silencing of *PFKFB3* gene was confirmed by immunoblotting.

### Western blot

Cells were washed in ice-cold PBS, lysed using RIPA buffer with protease inhibitors (Complete, Roche Diagnostics) and then centrifuged (250 × g, 5 min) to remove the debris. After measuring protein concentration by Bradford assay, samples containing 20 μg protein were mixed with 5 × loading buffer, denatured for 2 min at 95°C, loaded into a 12% polyacrylamide gel, run at 100 V for 2 h and then transferred onto a PVDF membrane (Millipore) for 3 h at 40 V. Next, the membrane was blocked with 5% non-fat dried milk powder (Applichem) in Tris-buffered saline (TBS)/ 0.1% Tween 20 (TBST) and then incubated with anti rabbit-PFKFB3 (Sigma), anti-beta-actin mAb (Abcam), anti-PFK-1 (H-55) (Santa Cruz Biotechnology) overnight at 4°C. The membrane was then washed 3 × 10 min with TBST and incubated with appropriate horseradish peroxidase–conjugated secondary antibodies for 1 h. The membrane was further washed 3 × 10 min with TBST before development using Amersham ECL Plus Western blotting developing kit (GE Technologies). Bands on membranes were visualized with a luminescence image analyzer (LAS 1000, Fuji Film) and analyzed with Image J software (available at http://rsb.info.nih.gov/ij; developed by Wayne Rasband, NIH).

### Quantitative reverse transcription polymerase chain reaction (RT- qPCR)

Total RNA was isolated from 0.5 × 10^6^ cells using the High Pure RNA Isolation Kit (Roche) according to the manufacturer's protocol and the concentration of total RNA was determined using a NanoDrop™ spectrophotometer (Thermo Scientific). For cDNA synthesis, 200 ng of total RNA was mixed with master mix from Maxima® First Strand cDNA Synthesis Kit for RT-qPCR (Thermo Scientific) and loaded into a thermal cycler (CFX96™ real-time PCR detection system, Biorad). Briefly, samples were incubated for 10 min at 25°C followed by 15 min at 50°C. The reaction was terminated by heating at 85°C for 5 min. Next, 2 μl of total cDNA was added to a 20 μl reaction mix containing iQ™ SYBR® Green Supermix (Biorad) and 200 nM of each primer. Triplicate reactions were performed foreach gene in a 96-well plate using a two-step amplificationprogram of initial denaturation at 95°C for 3 min, followed by 40 cycles of 95°C for 15 s and 60°C for 60 s using a CFX96™ real-time PCR detection system (Biorad).

The following primers were used:

PFKFB3 isoform 1:

5′-CTGCAGAGGAGATGCCCTAC-3′ and

5′-AGGTCCCTTCTTTGCATCCT-3′

PFKFB3 isoform 2:

5′-CACCGGGGAGTCCTACCA-3′ and

5′-TAGGGCATCTCCTCTGCACT-3′

PFK1:

5′-TGGGACTAAAAGGACTCTACCC-3′ and

5′-CCCTGTGTAAGCCTCAAAGC-3′

GAPDH:

5′-TCAACTACATGGTTTACATGTTC-3′ and

5′-GATCTCGCTCCTGGAAGAT-3′.

Melting curve analysis was applied to check the specificity of each PCR product and no nonspecific amplification or primer-dimerswere detected in any of the reactions. The relative quantification of gene expression was determined using the 2−ΔΔCT method [57]. Fold-changes in gene expression were assessed by a relative quantitation where the input amounts were normalized to an internal control gene (*GAPDH*), and a calibrator (non-treated cancer cells).

### ATP assay

SKBR3 cells, fibroblasts, and iPS cells grown with 10 mM 2-deoxy-D-glucose (Sigma-Aldrich) for 24 h were washed with ice-cold PBS and incubated with 100 μl of Somatic Cell ATP Releasing Agent (Sigma-Aldrich) for 5 min. Next, 50 μl of the cell extract was added into wells of a light-protected 96-well plate that was preloaded with 100 μl of ATP Assay Mix Solution (Sigma-Aldrich) and incubated for 3 min at RT. Subsequently, the luminescence was measured using a luminescence plate reader (VictorX4, PerkinElmer).

### Statistics

Unpaired (two-tailed) Student's *t*-tests with Welch's correction were performed for analysis of data. Values are shown as means ± SD. Results were considered to be significant when *P* was <0.05.

## SUPPLEMENTARY FIGURE



## Abbreviations

2-DG: 2-deoxy-D-glucose
3PO: 3-(3-pyridinyl)-1-(4-pyridinyl)-2-propen-1-one
AKT: protein kinase B
ALDH: aldehyde dehydrogenase
AP: alkaline phosphatase
APC: allophycocyanin
APC/C: anaphase promoting complex/cyclosome
ATP: adenosine 5′-triphosphate
CDK: cyclin dependent kinase
cDNA: complementary deoxyribonucleic acid
CSC: cancer stem cells
DAPI: 4′,6-diamidino-2-phenylindole
DEAB: diethylaminobenzaldehyde
DTB: double tymidine block
EDTA: ethylenediaminetetraacetic acid
ESC: embryonic stem cell
FACS: fluorescence activated cell sorting
F2,6BP: fructose-2,6-bisphosphate
GAPDH: glyceraldehyde 3-phosphate dehydrogenase
hEGF: human epidermal growth factor
HIF-1α: hypoxia-inducible factor 1-alpha
iPS: induced pluripotent stem cells
MEF: mouse embryonic fibroblasts
MK2: MAPK (mitogen-activated protein kinase)-activated protein kinase 2
MOI: multiplicity of infection
mRNA: messenger ribonucleic acid
mTOR: mammalian target of rapamycin
NT: non-targeting
PFK1: 6-phosphofructo-1-kinase
PFKFB3: 6-phosphofructo-2-kinase/fructose-2,6-biphosphatase, isoform 3
PI: propidium iodide
PI3K: phosphatidylinositol 3-kinase
PolyHEMA: poly-2-hydroxyethyl methacrylate
PS: pencillin/streptomycin
PVDF: polyvinylidene fluoride
R-point: restriction point
RT: room temperature
RT-qPCR: reverse transcription quantitative polymerase chain reaction
TNF-α: tumor necrosis factor alpha.

## References

[1] Takahashi K, Yamanaka S (2013). Induced pluripotent stem cells in medicine and biology. Development.

[2] Takahashi K, Tanabe K, Ohnuki M, Narita M, Ichisaka T, Tomoda K, Yamanaka S (2007). Induction of pluripotent stem cells from adult human fibroblasts by defined factors. Cell.

[3] Miura K, Okada Y, Aoi T, Okada A, Takahashi K, Okita K, Nakagawa M, Koyanagi M, Tanabe K, Ohnuki M, Ogawa D, Ikeda E, Okano H, Yamanaka S (2009). Variation in the safety of induced pluripotent stem cell lines. Nature biotechnology.

[4] Gutierrez-Aranda I, Ramos-Mejia V, Bueno C, Munoz-Lopez M, Real PJ, Macia A, Sanchez L, Ligero G, Garcia-Parez JL, Menendez P (2010). Human induced pluripotent stem cells develop teratoma more efficiently and faster than human embryonic stem cells regardless the site of injection. Stem cells.

[5] Ben-Porath I, Thomson MW, Carey VJ, Ge R, Bell GW, Regev A, Weinberg RA (2008). An embryonic stem cell-like gene expression signature in poorly differentiated aggressive human tumors. Nat Genet.

[6] Shats I, Gatza ML, Chang JT, Mori S, Wang J, Rich J, Nevins JR (2011). Using a stem cell-based signature to guide therapeutic selection in cancer. Cancer Res.

[7] Schoenhals M, Kassambara A, De Vos J, Hose D, Moreaux J, Klein B (2009). Embryonic stem cell markers expression in cancers. Biochemical and biophysical research communications.

[8] Nagata S, Hirano K, Kanemori M, Sun LT, Tada T (2012). Self-renewal and pluripotency acquired through somatic reprogramming to human cancer stem cells. PloS one.

[9] Wicha MS, Liu S, Dontu G (2006). Cancer stem cells: an old idea—a paradigm shift. Cancer Res.

[10] Reya T, Morrison SJ, Clarke MF, Weissman IL (2001). Stem cells, cancer, and cancer stem cells. Nature.

[11] Hombach-Klonisch S, Paranjothy T, Wiechec E, Pocar P, Mustafa T, Seifert A, Zahl C, Gerlach KL, Biermann K, Steger K, Hoang-Vu C, Schulze-Osthoff K, Los M (2008). Cancer stem cells as targets for cancer therapy: selected cancers as examples. Archivum immunologiae et therapiae experimentalis.

[12] Knoepfler PS (2009). Deconstructing stem cell tumorigenicity: a roadmap to safe regenerative medicine. Stem cells.

[13] Ghule PN, Medina R, Lengner CJ, Mandeville M, Qiao M, Dominski Z, Lian JB, Stein JL, Van Wijnen AJ, Stein GS (2011). Reprogramming the Pluripotent Cell Cycle: Restoration of an Abbreviated G1 Phase in Human Induced Pluripotent Stem (iPS) Cells. J Cell Physiol.

[14] Wasik AMGJ, Pantovic A, Cieślar-Pobuda A, Asgari HR, Bundgaard-Nielsen C, Rafat M, Dixon IMC, Ghavami S, Łos MJ (2014). Reprogramming and carcinogenesis - parallels and distinctions. Int Rev Cell Mol Biol.

[15] Harper LJ, Costea DE, Gammon L, Fazil B, Biddle A, Mackenzie IC (2010). Normal and malignant epithelial cells with stem-like properties have an extended G2 cell cycle phase that is associated with apoptotic resistance. BMC cancer.

[16] Baserga R (1994). Oncogenes and the strategy of growth factors. Cell.

[17] Campisi J, Medrano EE, Morreo G, Pardee AB (1982). Restriction point control of cell growth by a labile protein: evidence for increased stability in transformed cells. Proceedings of the National Academy of Sciences of the United States of America.

[18] Blagosklonny MV, Pardee AB (2002). The restriction point of the cell cycle. Cell cycle.

[19] Colombo SL, Palacios-Callender M, Frakich N, Carcamo S, Kovacs I, Tudzarova S, Moncada S (2011). Molecular basis for the differential use of glucose and glutamine in cell proliferation as revealed by synchronized HeLa cells. Proc Natl Acad Sci U S A.

[20] Tudzarova S, Colombo SL, Stoeber K, Carcamo S, Williams GH, Moncada S (2011). Two ubiquitin ligases, APC/C-Cdh1 and SKP1-CUL1-F (SCF)-beta-TrCP, sequentially regulate glycolysis during the cell cycle. Proc Natl Acad Sci U S A.

[21] Jang M, Kim SS, Lee J (2013). Cancer cell metabolism: implications for therapeutic targets. Exp Mol Med.

[22] Caro P, Kishan AU, Norberg E, Stanley IA, Chapuy B, Ficarro SB, Polak K, Tondera D, Gounarides J, Yin H, Zhou F, Green MR, Chen L, Monti S, Marto JA, Shipp MA (2012). Metabolic signatures uncover distinct targets in molecular subsets of diffuse large B cell lymphoma. Cancer cell.

[23] Marin-Valencia I, Yang C, Mashimo T, Cho S, Baek H, Yang XL, Rajagopalan KN, Maddie M, Vemireddy V, Zhao Z, Cai L, Good L, Tu BP, Hatanpaa KJ, Mickey BE, Mates JM (2012). Analysis of tumor metabolism reveals mitochondrial glucose oxidation in genetically diverse human glioblastomas in the mouse brain *in vivo*. Cell Metab.

[24] Warburg O (1956). On the origin of cancer cells. Science.

[25] Marie SK, Shinjo SM (2011). Metabolism and brain cancer. Clinics (Sao Paulo).

[26] Yalcin A, Telang S, Clem B, Chesney J (2009). Regulation of glucose metabolism by 6-phosphofructo-2-kinase/fructose-2, 6-bisphosphatases in cancer. Experimental and molecular pathology.

[27] Kessler R, Bleichert F, Warnke JP, Eschrich K (2008). 6-Phosphofructo-2-kinase/fructose-2,6-bisphosphatase (PFKFB3) is up-regulated in high-grade astrocytomas. Journal of neuro-oncology.

[28] Novellasdemunt L, Obach M, Millan-Arino L, Manzano A, Ventura F, Rosa JL, Jordan A, Navarro-Sabate A, Bartrons R (2012). Progestins activate 6-phosphofructo-2-kinase/fructose-2,6-bisphosphatase 3 (PFKFB3) in breast cancer cells. Biochem J.

[29] Calvo MN, Bartrons R, Castano E, Perales JC, Navarro-Sabate A, Manzano A (2006). PFKFB3 gene silencing decreases glycolysis, induces cell-cycle delay and inhibits anchorage-independent growth in HeLa cells. FEBS letters.

[30] Klarer AC, O'Neal J, Imbert-Fernandez Y, Clem A, Ellis SR, Clark J, Clem B, Chesney J, Telang S (2014). Inhibition of 6-phosphofructo-2-kinase (PFKFB3) induces autophagy as a survival mechanism. Cancer & metabolism.

[31] Pardee AB (1989). G1 events and regulation of cell proliferation. Science.

[32] Klonisch T, Wiechec E, Hombach-Klonisch S, Ande SR, Wesselborg S, Schulze-Osthoff K, Los M (2008). Cancer stem cell markers in common cancers - therapeutic implications. Trends in molecular medicine.

[33] Ginestier C, Hur MH, Charafe-Jauffret E, Monville F, Dutcher J, Brown M, Jacquemier J, Viens P, Kleer CG, Liu S, Schott A, Hayes D, Birnbaum D, Wicha MS, Dontu G (2007). ALDH is a marker of normal and malignant human mammary stem cells and a predictor of poor clinical outcome. Cell stem cell.

[34] Ghebeh H, Sleiman GM, Manogaran PS, Al-Mazrou A, Barhoush E, Al-Mohanna FH, Tulbah A, Al-Faqeeh K, Adra CN (2013). Profiling of normal and malignant breast tissue show CD44 high/CD24low phenotype as a predominant stem/progenitor marker when used in combination with Ep-CAM/CD49f markers. BMC cancer.

[35] Al-Hajj M, Wicha MS, Benito-Hernandez A, Morrison SJ, Clarke MF (2003). Prospective identification of tumorigenic breast cancer cells. Proceedings of the National Academy of Sciences of the United States of America.

[36] Fillmore CM, Kuperwasser C (2008). Human breast cancer cell lines contain stem-like cells that self-renew, give rise to phenotypically diverse progeny and survive chemotherapy. Breast cancer research: BCR.

[37] Yoshida Y, Takahashi K, Okita K, Ichisaka T, Yamanaka S (2009). Hypoxia enhances the generation of induced pluripotent stem cells. Cell stem cell.

[38] Minchenko O, Opentanova I, Minchenko D, Ogura T, Esumi H (2004). Hypoxia induces transcription of 6-phosphofructo-2-kinase/fructose-2,6-biphosphatase-4 gene via hypoxia-inducible factor-1alpha activation. FEBS letters.

[39] Clem B, Telang S, Clem A, Yalcin A, Meier J, Simmons A, Rasku MA, Arumugam S, Dean WL, Eaton J, Lane A, Trent JO, Chesney J (2008). Small-molecule inhibition of 6-phosphofructo-2-kinase activity suppresses glycolytic flux and tumor growth. Mol Cancer Ther.

[40] Bando H, Atsumi T, Nishio T, Niwa H, Mishima S, Shimizu C, Yoshioka N, Bucala R, Koike T (2005). Phosphorylation of the 6-phosphofructo-2-kinase/fructose 2,6-bisphosphatase/PFKFB3 family of glycolytic regulators in human cancer. Clinical cancer research: an official journal of the American Association for Cancer Research.

[41] Atsumi T, Chesney J, Metz C, Leng L, Donnelly S, Makita Z, Mitchell R, Bucala R (2002). High expression of inducible 6-phosphofructo-2-kinase/fructose-2,6-bisphosphatase (iPFK-2, PFKFB3) in human cancers. Cancer Res.

[42] Duran J, Obach M, Navarro-Sabate A, Manzano A, Gomez M, Rosa JL, Ventura F, Perales JC, Bartrons R (2009). Pfkfb3 is transcriptionally upregulated in diabetic mouse liver through proliferative signals. The FEBS journal.

[43] Almeida A, Bolanos JP, Moncada S (2010). E3 ubiquitin ligase APC/C-Cdh1 accounts for the Warburg effect by linking glycolysis to cell proliferation. Proc Natl Acad Sci U S A.

[44] Becker KA, Stein JL, Lian JB, van Wijnen AJ, Stein GS (2010). Human embryonic stem cells are pre-mitotically committed to self-renewal and acquire a lengthened G1 phase upon lineage programming. Journal of cellular physiology.

[45] Mannello F (2013). Understanding breast cancer stem cell heterogeneity: time to move on to a new research paradigm. BMC Med.

[46] Telang S, Yalcin A, Clem AL, Bucala R, Lane AN, Eaton JW, Chesney J (2006). Ras transformation requires metabolic control by 6-phosphofructo-2-kinase. Oncogene.

[47] Mor I, Cheung EC, Vousden KH (2011). Control of glycolysis through regulation of PFK1: old friends and recent additions. Cold Spring Harb Symp Quant Biol.

[48] Obach M, Navarro-Sabate A, Caro J, Kong X, Duran J, Gomez M, Perales JC, Ventura F, Rosa JL, Bartrons R (2004). 6-Phosphofructo-2-kinase (pfkfb3) gene promoter contains hypoxia-inducible factor-1 binding sites necessary for transactivation in response to hypoxia. The Journal of biological chemistry.

[49] Lu H, Forbes RA, Verma A (2002). Hypoxia-inducible factor 1 activation by aerobic glycolysis implicates the Warburg effect in carcinogenesis. J Biol Chem.

[50] Bobarykina AY, Minchenko DO, Opentanova IL, Moenner M, Caro J, Esumi H, Minchenko OH (2006). Hypoxic regulation of PFKFB-3 and PFKFB-4 gene expression in gastric and pancreatic cancer cell lines and expression of PFKFB genes in gastric cancers. Acta Biochim Pol.

[51] Kawano Y, Kikukawa Y, Fujiwara S, Wada N, Okuno Y, Mitsuya H, Hata H (2013). Hypoxia reduces CD138 expression and induces an immature and stem cell-like transcriptional program in myeloma cells. International journal of oncology.

[52] Muz B, de la Puente P, Azab F, Luderer M, Azab AK (2014). Hypoxia promotes stem cell-like phenotype in multiple myeloma cells. Blood Cancer J.

[53] Heddleston JM, Li Z, McLendon RE, Hjelmeland AB, Rich JN (2009). The hypoxic microenvironment maintains glioblastoma stem cells and promotes reprogramming towards a cancer stem cell phenotype. Cell cycle.

[54] Telang S, Clem BF, Klarer AC, Clem AL, Trent JO, Bucala R, Chesney J (2012). Small molecule inhibition of 6-phosphofructo-2-kinase suppresses t cell activation. J Transl Med.

[55] Sweet S, Singh G (1995). Accumulation of human promyelocytic leukemic (HL-60) cells at two energetic cell cycle checkpoints. Cancer Res.

[56] Rakar J, Lonnqvist S, Sommar P, Junker J, Kratz G (2012). Interpreted gene expression of human dermal fibroblasts after adipo-, chondro- and osteogenic phenotype shifts. Differentiation; research in biological diversity.

[57] Livak KJ, Schmittgen TD (2001). Analysis of relative gene expression data using real-time quantitative PCR and the 2(-Delta Delta C(T)) Method. Methods.

