# Supplementary material for: The expression pattern of PFKFB3 enzyme distinguishes between induced-pluripotent stem cells and cancer stem cells
Source: Oncotarget. 2015 Aug 13;6(30):29753–70. doi: 10.18632/oncotarget.4995 (PMC4745760; doi:10.18632/oncotarget.4995)
Supplement: Supplementary file 1 [file oncotarget-06-29753-s001.pdf]

SUPPLEMENTARY FIGURE

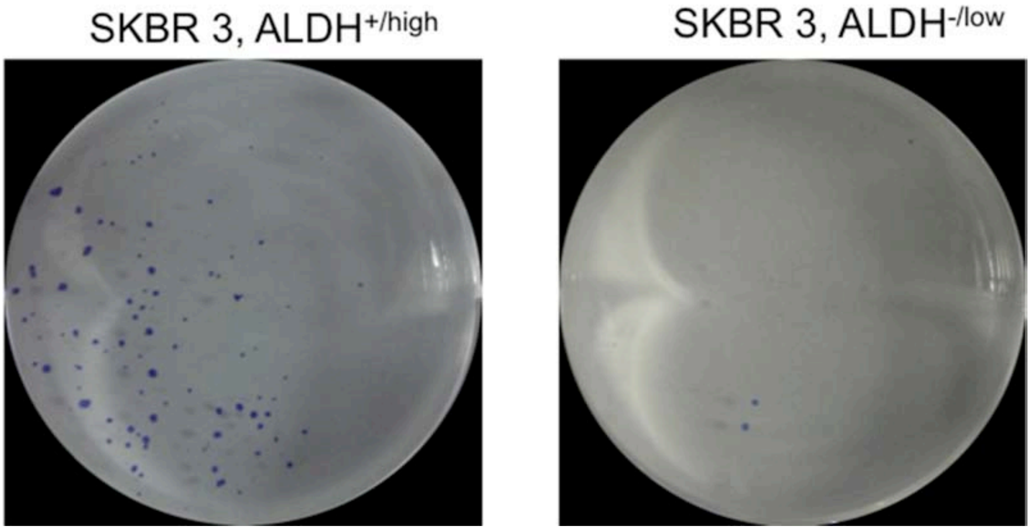

Supplementary Figure S1: Colony formation by SKBR3 ALDH^{high} and SKBR3 ALDH^{low} cells. ALDH^{high} and ALDH^{low} cells were isolated from the SKBR3 cell line, plated on a soft agar surface, cultured for 2–3 weeks and stained with crystal violet. A representative plate showing an increased number of SKBR3 ALDH^{high} cells compared to SKBR3 ALDH^{low} is presented.
